# The complete chloroplast genome sequence of *Bambusa tuldoides* f. *swolleninternode* (Poaceae: Bambuseae)

**DOI:** 10.1080/23802359.2023.2181648

**Published:** 2023-02-24

**Authors:** Ben Wang, Bo Qin, Kaidao Sun, Xin Huang, Shangbin Bai, Xiumei Zhou

**Affiliations:** a College of Urban and Rural Construction, Shanxi Agricultural University, Jinzhong, China; b Guangxi Key Laboratory of Special Non-wood Forest Cultivation & Utilization, Guangxi Forestry Research Institute, Nanning, China; c Jiyang College, Zhejiang A&F University, Zhuji, China

**Keywords:** Chloroplast genome, *Bambusa tuldoides* f. *swolleninternode*, phylogenetic analysis

## Abstract

*Bambusa tuldoides* f. *swolleninternode* is an attractive ornamental bamboo species of southern China, with highly shortened and swollen at the base of internodes. In this study, the complete chloroplast genome of *B. tuldoides* was sequenced and reported for the first time. The complete genome size is 139,460 base pairs (bp), including a large single-copy (LSC) region of 82,996 bp, a small single-copy (SSC) region of 12,876 bp and a pair of invert repeats (IR) regions of 21,794 bp. The plastid genome contained 132 genes, including 86 protein-coding genes, 38 tRNA genes and 8 rRNA genes. The overall GC content of the genome is 39%. The phylogenetic analysis revealed that *B. tuldoides* is closely related to *B. dolichoclada*, *B. pachinensis* var. *hirsutissima*, and *B. utilis,* three species in *Bambusa* based on 16 chloroplast genomes.

## Introduction

With extremely shortened and swollen at the base of internodes, *Bambusa tuldoides* f. *swolleninternode* N. H. Xia (Xia et al. 1996) is an important landscape resource (http://www.iplant.cn/info/bambusa%20tuldoides%20f.%20swolleninternode?t=z), and is mainly distributed in the Guangdong province, China. At present, researches on *B. tuldoides* mainly focus on the photosynthetic and chlorophyll fluorescence characteristics, the activity of soil enzymes, dust retention, and resistance to environment stress aspects (Long 2017). Due to the conserved genome structure and relatively high substitution rate of the chloroplast genome, obtaining chloroplast genome information will provide valuable information for relevant research, such as species identification, genetics, and phylogeny (Daniell et al. 2021; Li et al. 2021). To better understand the taxonomic and evolutionary relationship of *Bambusa*, we assembled the complete chloroplast genome of *B. tuldoides* based on Illumina pair-end sequencing data in this study.

## Materials and methods

**Figure 1.**
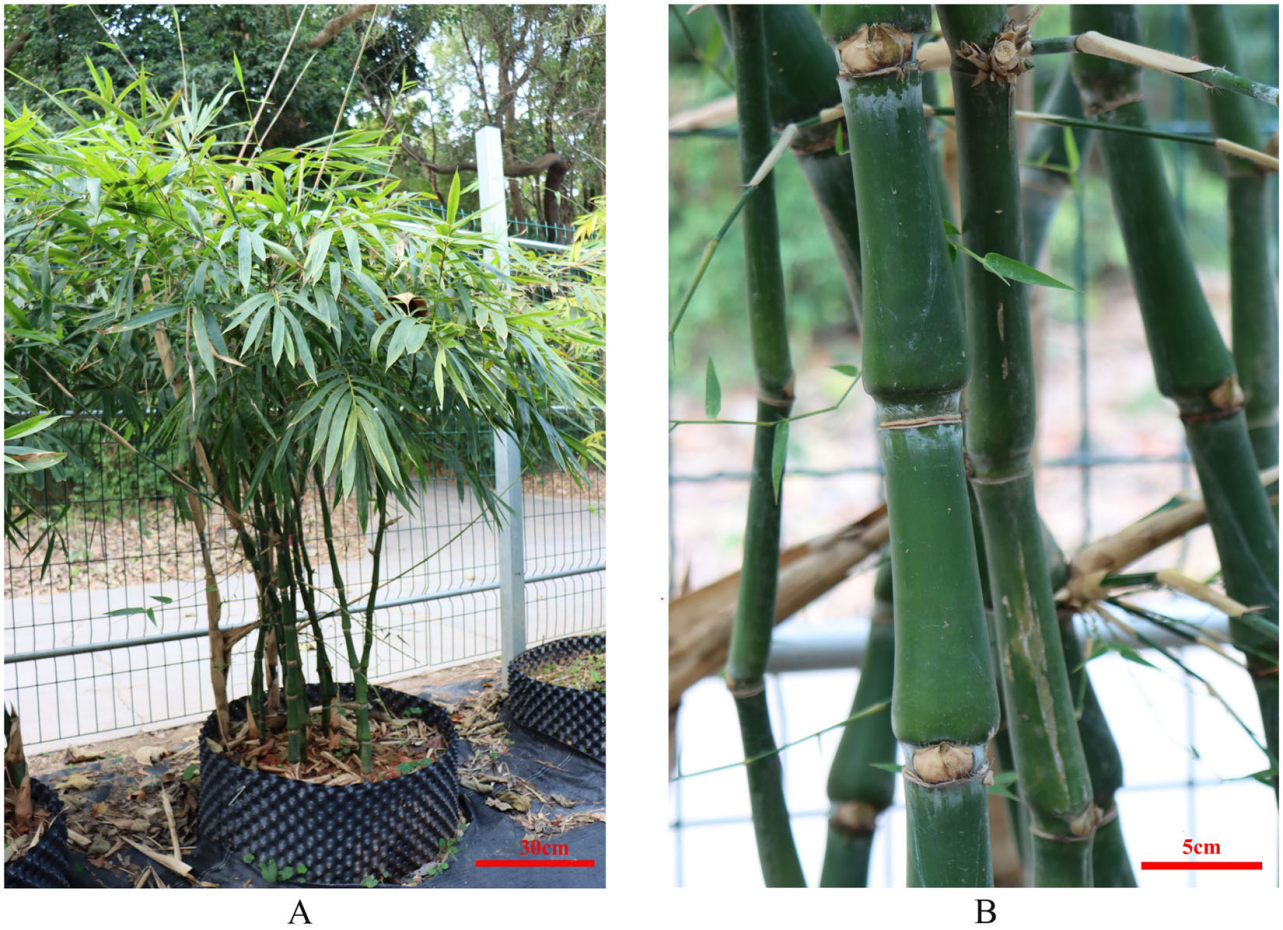
The reference image of the plant of *Bambusa tuldoides* f. *swolleninternode* (taken by the authors; A: whole plant; B: aboveground stems of plant).

**Figure 2.**
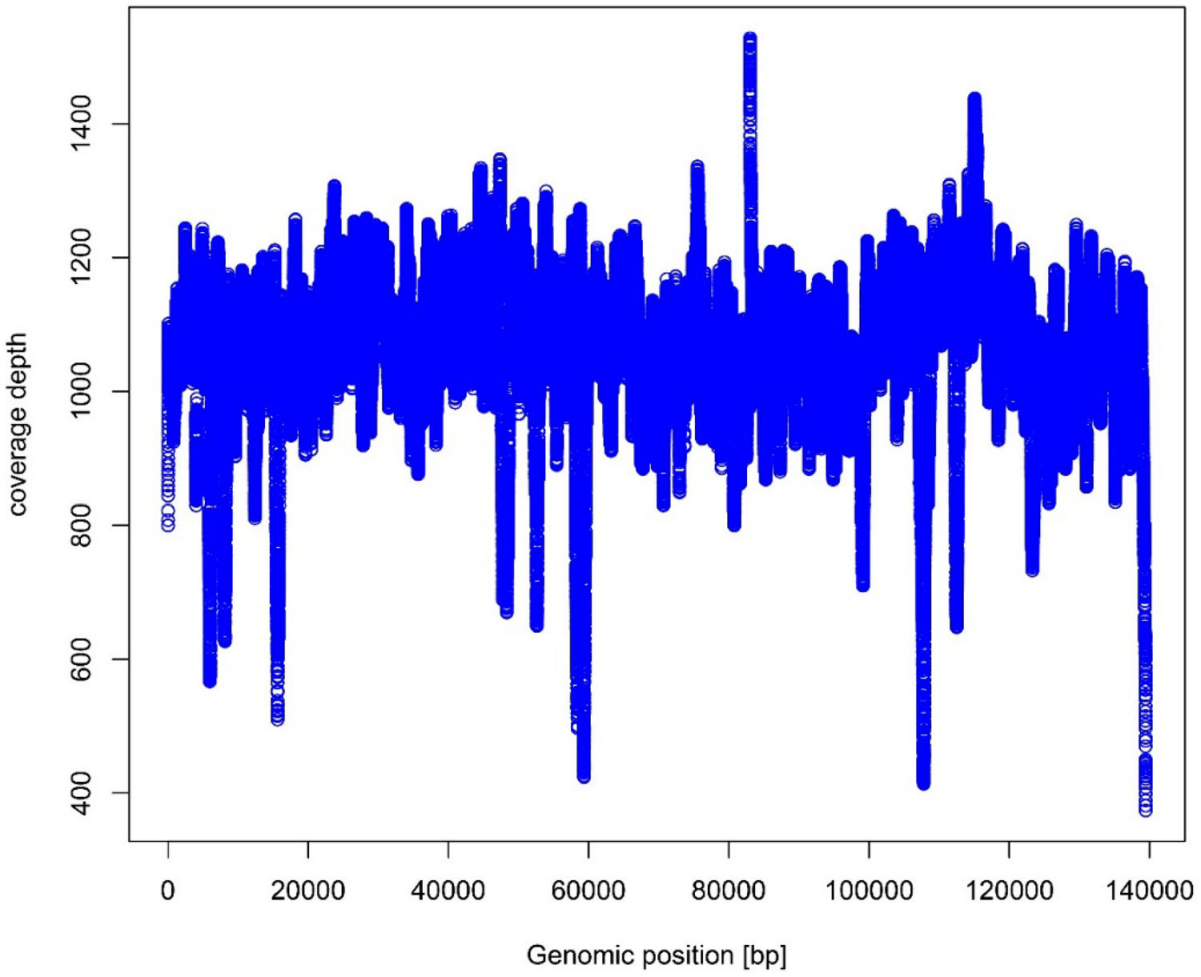
The read coverage depth map of the assembly of the chloroplast genome of *Bambusa tuldoides* f. *swolleninternode* (the figure means the coverage depth on each base of chloroplast genome of *Bambusa tuldoides* f. *swolleninternode*; X-axis indicates the location of bases in the genome; Y-axis indicates the coverage depth).

The sample of *B. tuldoides* selected for this study was located in Nanning, Guangxi Province, China (116°41′N, 39°91′E, Figure 1), in the Bamboo Germplasm Resource Garden of Guangxi Forestry Research Institute. The certificate specimens were deposited in the herbarium of Guangxi Forestry Research Institute (http://www.gxlky.com.cn/, Mr. Li, email: zzcx_gfri@163.com) under the registration number of 20220407. Fresh leaves of *B. tuldoides* were collected for the preparation of genomic DNA extraction, which were then frozen by liquid nitrogen and stored at −80 °C. Total genomic DNA was extracted using the Doyle’s (1987) method. The PE reads of 150 bp were generated by the platform of Illumina NovaSeq 6000 (Illumina, San Diego, CA, USA). The chloroplast genome was assembled using the SPAdes v3.14.1 software (Bankevich et al. 2012; Shen et al. 2019; Zheng et al. 2020). A total number of 4.23 G raw data were analyzed to generate 4.18 G clean data with the error rate 0.03 after quality control processing, and reached 1064Χ coverage over the chloroplast genome (Figure 2). The chloroplast genome annotation was performed using PGA program (Qu et al. 2019), using the *Bambusa dolichoclada* (GenBank accession NC_063133.1) chloroplast genome as the reference. *Indocalamus wilsonii* and *Fargesia dracocephala* were selected as outgroups. Sixteen plastid genomes of *Bambusa* related to *B. tuldoides* were chosen to draw the phylogenetic tree. The sequences used in this study were downloaded from NCBI GenBank. The 71 common protein-coding genes in each complete chloroplast genome of 16 species were aligned with the genes in *B. tuldoides* using MAFFT 7.037 (Katoh and Standley 2016) with the FFT-NS-2 strategy. Then, the best fitting model, JTT + F + R2 model, was screened by running the model-finder for 1.6 (Kalyaanamoorthy et al. 2017). Finally, iqtree 2.0 was used to construct a phylogenetic tree with 1,000 bootstraps based on the ML method.

## Results

**Figure 3.**
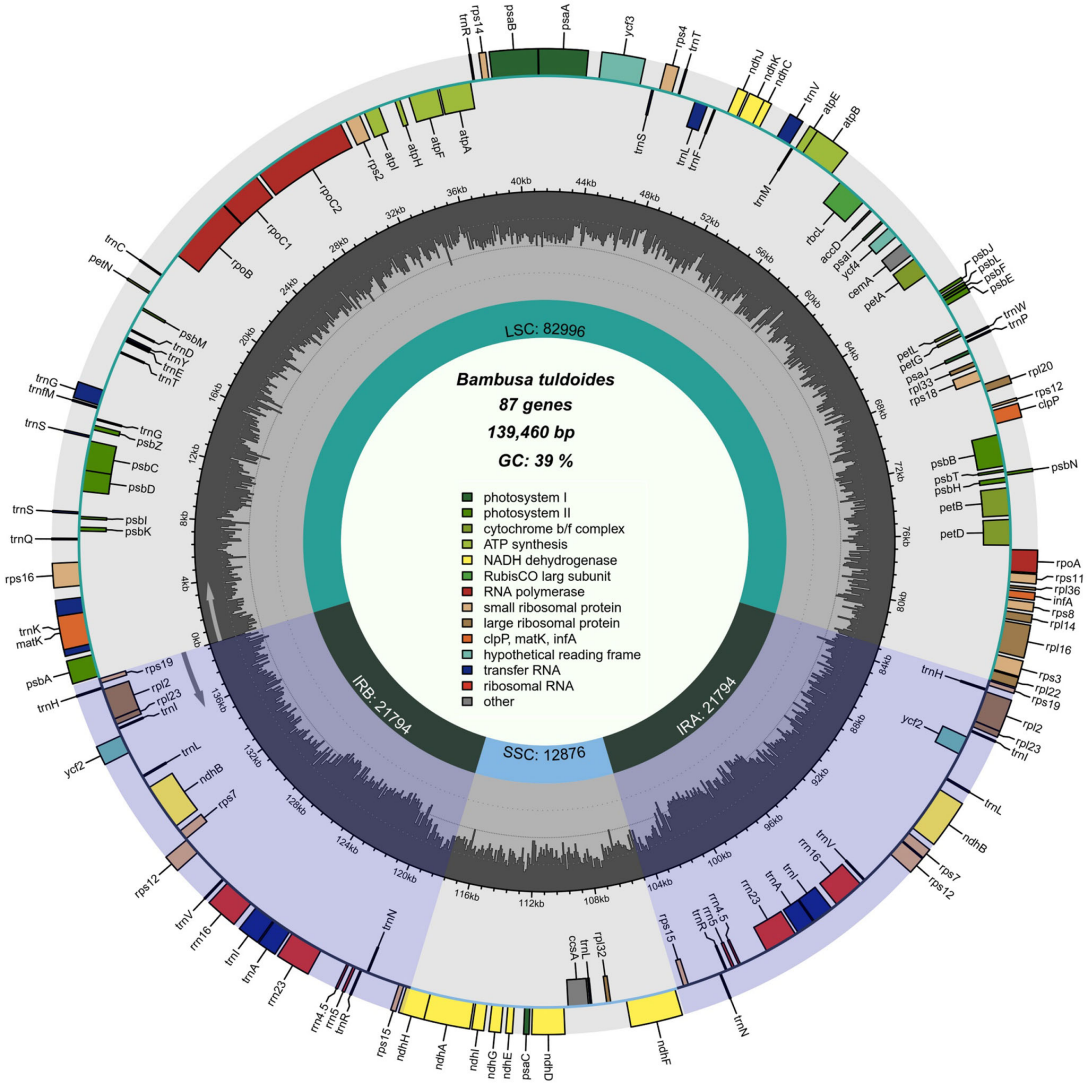
Chloroplast genome map of *Bambusa tuldoides* f. *swolleninternode*. The purple areas indicate the extent of the inverted repeats (IRA and IRB), which separate the genome into small (SSC) and large (LSC) single copy regions. Genes reside on the inside and outside of the outer circle are in the forward and reverse directions, respectively. Boxes on the outside and inside of the outer circle respectively represent genes transcribed clockwise and anti-clockwise. The dark and light gray bars in the inner circle denote G + C and A + T contents, respectively.

A total of 14,089,624 raw reads of chloroplast genome of *B. tuldoides* (GenBank number: OM687229) was deposited to the NCBI Sequence Read Archive (SRA: SRR21700304). The complete chloroplast genome of *B. tuldoides* is 139,460 bp in length, containing four typical regions, a large single-copy region (LSC, 82,996 bp), a small single-copy region (SSC, 12,876 bp) and two identical inverted repeat regions (IR, 21,794 bp). The overall GC content of chloroplast genome of *B. tuldoides* is 39%. The chloroplast genome contains 132 functional genes including: 8 rRNA genes, 38 tRNA genes and 86 protein-coding genes (Figure 3). Some genes are difficult to annotate, including trans- and cis-splicing genes. It includes 19 Cis-splicing genes (including *atpF, ndhA, ndhB, petB, petD, rpl2, rpl16, rps16, trnA-UGC, trnG-UCC, trnI-GAU, trnK-UUU, trnL-UAA, trnV-UAC,* and *ycf3;* in which *ndhB, rpl2, trnA-UGC and trnI-GAU* four genes exist two copies) and one Trans-splicing gene (*rps12*). One of the Cis-splicing gene (*ycf3*) contains two introns, the other genes contain one intron. The structure of the 12 protein-coding trans- and cis-splicing genes had been shown in Supplement Figure S1.

**Figure 4.**
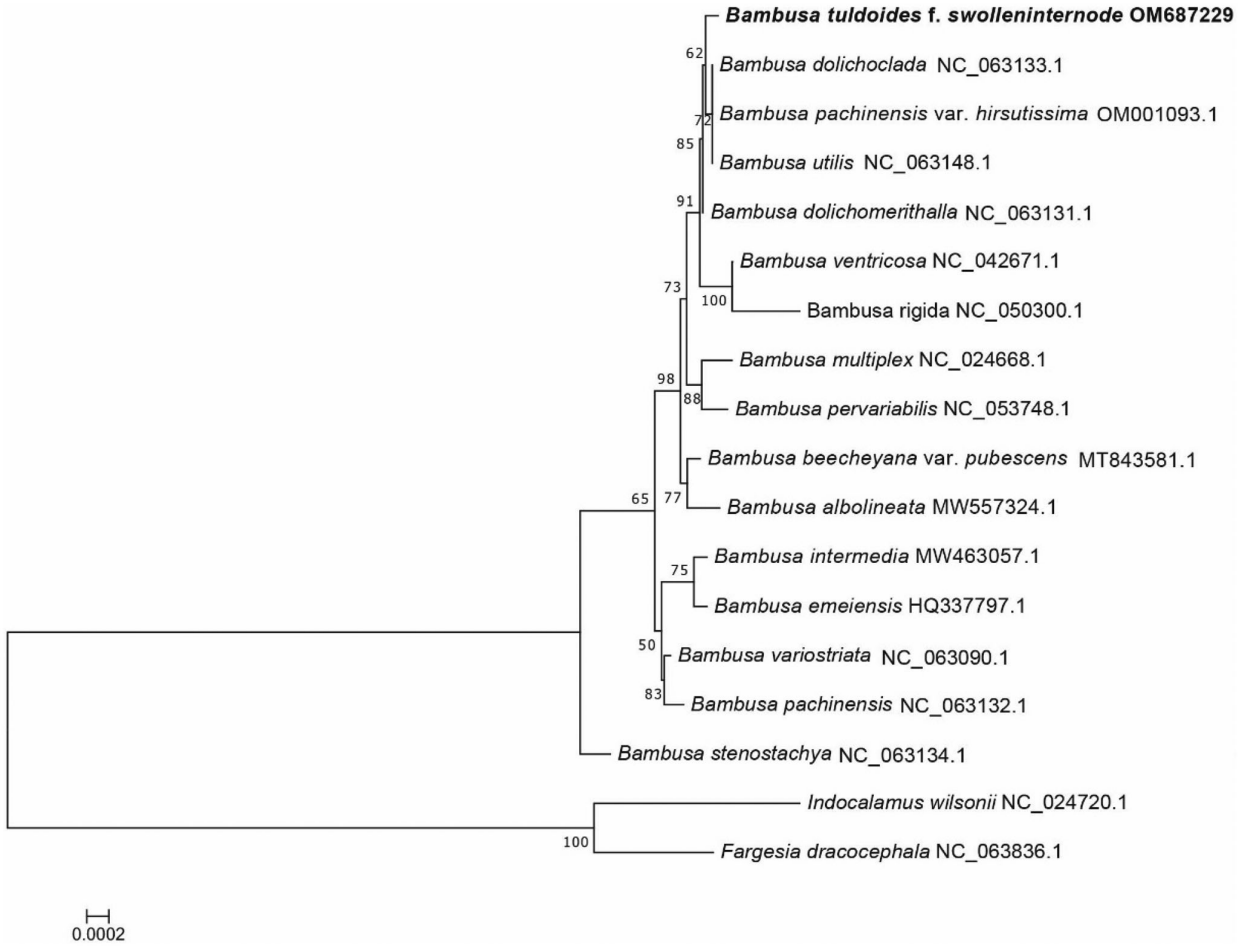
Phylogenetic tree of 16-taxon data of *Bambusa* set based on 71 chloroplast protein-coding genes using best-fit and JTT + F+R2 model. *Indocalamus wilsonii* and *Fargesia dracocephala* were selected as outgroups.

The phylogenetic relationships of *B. tuldoides* with other members of *Bambusa* were explored, the results showed that the chloroplast genome of *B. tuldoides* is closely related to three species, including *B. dolichoclada*, *B. pachinensis* var. *hirsutissima*, and *B. utilis* (Figure 4). The analysis of the complete chloroplast genome of *B. tuldoides* will provide fundamental information for conservation, utilization and phylogenomic studies of *Bambusa.*

## Supplementary Material

Supplemental Material

## Data Availability

The genome sequence data that support the findings of this study are openly available in GenBank of the NCBI under the accession number of OM687229 (https://www.ncbi.nlm.nih.gov/nuccore/OM687229.1/). The associated BioProject, SRA, and Bio-Sample numbers are PRJNA884087, SRR21700304, and SAMN31008065, respectively.
