# Supplementary material for: The complete chloroplast genome sequence of Bambusa tuldoides f. swolleninternode (Poaceae: Bambuseae)
Source: Mitochondrial DNA B Resour. 2023 Feb 24;8(2):324–8. doi: 10.1080/23802359.2023.2181648 (PMC9970254; doi:10.1080/23802359.2023.2181648)
Supplement: Supplemental Material [file TMDN_A_2181648_SM7284.docx]

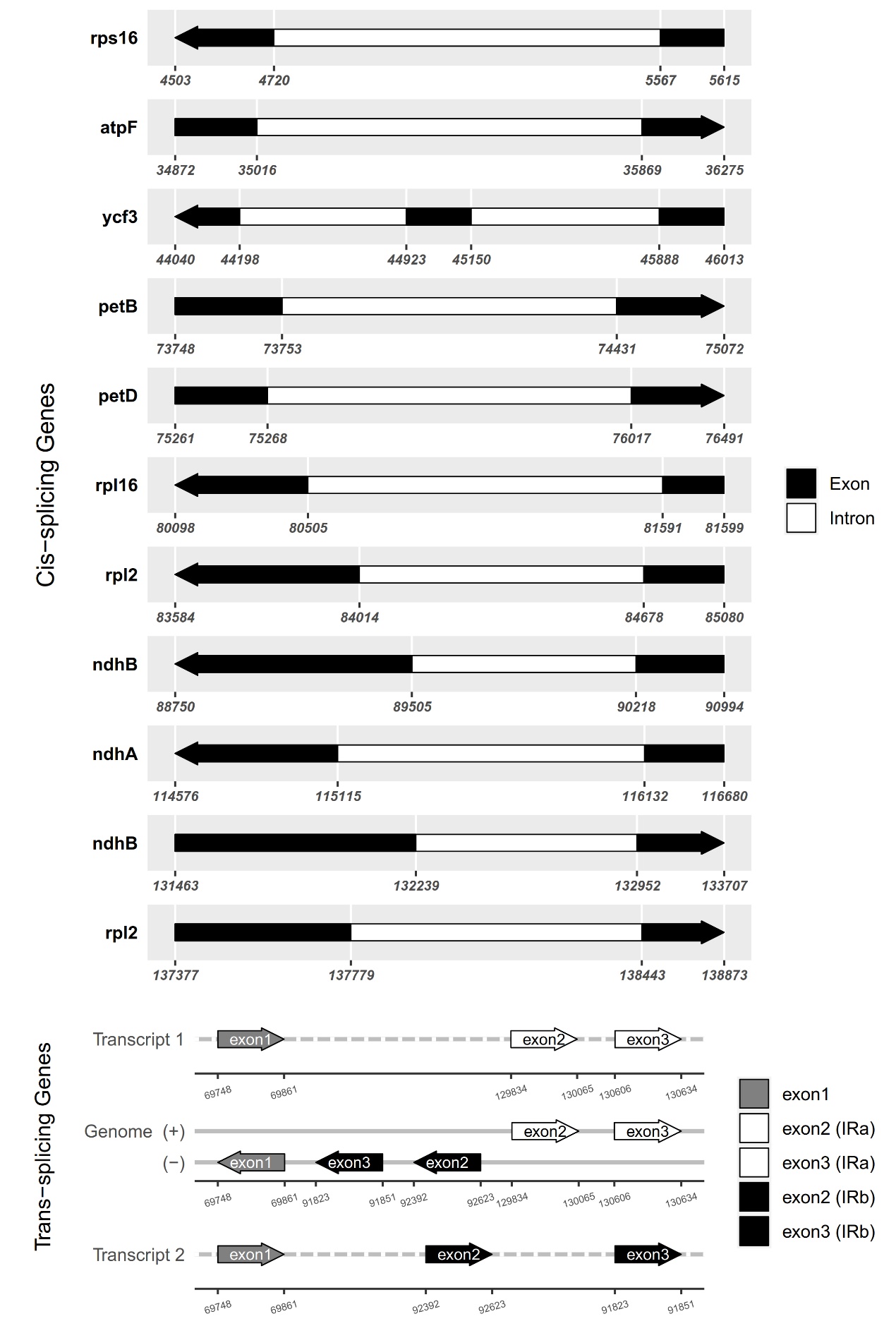
Supplement Figure S1 The structure of the 12 protein-coding trans- and cis-splicing genes annotated in Cp genome of *Bambusa tuldoides* f. *swolleninternode*. (including 11 cis-splicing genes and one trans-splicing gene *ycf3*; The numbers in the picture represent the location in the Cp genome of *Bambusa tuldoides* f. *swolleninternode*)
